# Chronic pain as an emergent property of a complex system and the potential roles of psychedelic therapies

**DOI:** 10.3389/fpain.2024.1346053

**Published:** 2024-04-19

**Authors:** Maya Armstrong, Joel Castellanos, Devon Christie

**Affiliations:** ^1^ Department of Family & Community Medicine, University of New Mexico, Albuquerque, NM, United States; ^2^ Division of Pain Medicine, Department of Anesthesiology, University of California, San Diego, CA, United States; ^3^ Department of Medicine, University of British Columbia, Vancouver, BC, Canada

**Keywords:** chronic pain, complexity, systems theory, biopsychosocial, neuroplasticity, psychedelics, mindfulness

## Abstract

Despite research advances and urgent calls by national and global health organizations, clinical outcomes for millions of people suffering with chronic pain remain poor. We suggest bringing the lens of complexity science to this problem, conceptualizing chronic pain as an emergent property of a complex biopsychosocial system. We frame pain-related physiology, neuroscience, developmental psychology, learning, and epigenetics as components and mini-systems that interact together and with changing socioenvironmental conditions, as an overarching complex system that gives rise to the emergent phenomenon of chronic pain. We postulate that the behavior of complex systems may help to explain persistence of chronic pain despite current treatments. From this perspective, chronic pain may benefit from therapies that can be both disruptive and adaptive at higher orders within the complex system. We explore psychedelic-assisted therapies and how these may overlap with and complement mindfulness-based approaches to this end. Both mindfulness and psychedelic therapies have been shown to have transdiagnostic value, due in part to disruptive effects on rigid cognitive, emotional, and behavioral patterns as well their ability to promote neuroplasticity. Psychedelic therapies may hold unique promise for the management of chronic pain.

## 1 Introduction

Pain is a complex and dynamic response, defined as “an unpleasant sensory and emotional experience associated with, or resembling that associated with, actual or potential tissue damage.” This widely recognized definition was put forth by the International Association for the Study of Pain [initially in 1979 (1) and recently revised by Raja and colleagues (2)]. Pain can be acute, representing an immediate and short-term response to a specific event such as illness or injury, or it can become chronic. Both are complex, yet each has unique features and often respond differently to management interventions.

Acute pain generally is thought to be “informative” in that sensory information from an injured part of the body (e.g., a twisted ankle) sends a message to the brain that is interpreted as dangerous (i.e., tissue damage). The brain signals responses in the motor cortex and areas involved in planning and execution, resulting in adaptive, compensatory behaviors to reduce pain and ultimately facilitate a return to a “normal” or homeostatic state.

Pain that persists or repeatedly recurs for at least 3 or 6 months (depending on the definition) is considered chronic and generally can be categorized as nociceptive, neuropathic, or nociplastic. Nociceptive pain often is synonymous with tissue damage or pain related to inflammation and is considered a normal response to an intact somatosensory system (3). Neuropathic pain is a clinical entity related to a demonstrable lesion or disease specific to neuronal tissue (3). Nociplastic pain is the most recently recognized category; it refers to pain that persists in the absence of identifiable tissue damage or after any initial damage has resolved (3–5). Compared with acute pain, these chronic pain experiences are far more complex and less understood. Importantly, the sequelae of chronic pain are significant not only for individuals but for societies at large, as it is one of the leading reasons for disability worldwide (6–8). The toll of personal suffering as well as the tremendous direct and indirect costs associated with chronic pain syndromes have led to an impressive amount of research yet only minimal improvements in clinical outcomes (9, 10).

The unacceptably poor state of chronic pain management was the topic of a 2021 Lancet editorial, titled Rethinking Chronic Pain. The authors state: “Thinking on chronic pain needs to be reset to help patients understand their pain, shift expectations, and set realistic, individualized goals that prioritize function and quality of life, rather than complete pain relief” (11). This is not such a novel concept in the management of chronic conditions, such as diabetes, but has been slow to be adapted and adopted in the world of chronic pain management—a fact that has been lamented by many thought leaders in the field (4, 12–16). In this paper, we suggest conceptualizing chronic pain from a systems perspective to progress understanding and develop novel treatment strategies. Specifically, we discuss chronic pain as an emergent property of a complex system. Within complex systems, destabilizing perturbations can support assimilation of new information and hence new emergent phenomena. We propose that psychedelic-assisted therapies may create such “destabilization” within a carefully designed context that provides optimal new inputs. This may support the emergence of improved clinical and functional outcomes for patients experiencing chronic pain.

The concept of the “neuromatrix of pain” was introduced in the late 1980s by Canadian psychologist, Ronald Melzack, to explain the phenomenon of phantom limb pain (17), a condition wherein the supposed source of pain—a limb that had been traumatically injured and amputated—is no longer connected to the body. Melzack proposed that pain is generated not by peripheral tissues and sensory neurons but by several tiers of neural networks involving multiple brain structures. Information processed by these networks includes not only sensory and motor data but also emotional, cognitive, motivational, and relational data (17, 18).

Melzack's neuromatrix later evolved into the “pain matrix,” which categorized the neural circuits involved into three major hierarchical pathways reflecting three different dimensions of pain: primary sensory–discriminative, secondary motivational–affective, and tertiary evaluative dimensions (18). More recently, much attention has been paid to higher network-level interactions, constituting a “fourth order” of the pain matrix and an additional level of complexity. This includes the default mode network (DMN), the salience network (SN) and the central executive network, also known as the frontoparietal network (CEN/FPN). These networks are the focus of much research in a variety of neuropsychiatric conditions and are described extensively elsewhere (19–22). The DMN is often simplistically described as generating one's “resting mental state.” It is involved in self-referential thought and goal-independent activity (e.g., mind-wandering). The SN detects and responds to novel, relevant (i.e., salient) stimuli. The CEN/FPN is an executive network involved in executing goal-oriented activities.

The pain matrix also includes elements beyond neurons and neural networks, including the following cells:

Neuroendocrine cells: The hypothalamic-pituitary-adrenal (HPA) axis, comprised of neuroendocrine cells, is critically involved with response to stress, illness, and injury. Acute pain and acute stress often occur together (e.g., traumatic injury). After the initial sympathetic adrenal medullary response involving adrenaline and noradrenaline, the HPA stimulates neuroendocrine cells of the adrenal cortex to release glucocorticoids (e.g., cortisol in humans), which have many short- and long-term effects (23–25). An important long-term effect of cortisol is its influence on learning, which can contribute to the chronification of pain (26).

Immune cells: Microglia are unique, brain-resident macrophages, which appear to be critical regulators of neuronal function and behavior (27, 28). Initially believed only to have “housekeeping” duties, recent research has uncovered paradoxical roles in both neuroinflammation and neuroprotection (29). Like other macrophages, they can migrate to sites of damage (30). Importantly, the microenvironment, which includes neuro-immune information sharing in the form of cytokines and chemokines, influences the activation state and behavior of microglia. Depending on various microenvironmental influences, activated microglia can polarize into pro-inflammatory (M1) or anti-inflammatory (M2) states (31). Emerging data suggest that microglia—especially in their “activated” proinflammatory state—contribute to neuropathic pain (31, 32) and headache syndromes (33, 34) as well as treatment-resistant depression (35, 36) and other chronic or relapsing/remitting conditions. Activated microglia release neurotrophic factors that affect neuronal excitability (37, 38), long-term potentiation (39), and synaptic efficacy (40). Studies in animal models are helping to elucidate the activity of microglia in the spinal cord, which appears to be especially important in neuropathic pain (31). As laboratory and imaging techniques continue to advance, our understanding of the roles of brain-resident microglia in chronic pain syndromes will undoubtedly improve. Another type of immune cell, mast cells, typically associated with allergic reactions, also exist within the central nervous system and appear to have roles in neuroinflammation and pain (41), perhaps in part by affecting the microenvironment that then directs the activation and behavior of microglia.

## 2 Chronic pain as an emergent property of a complex system

**Figure 1:**
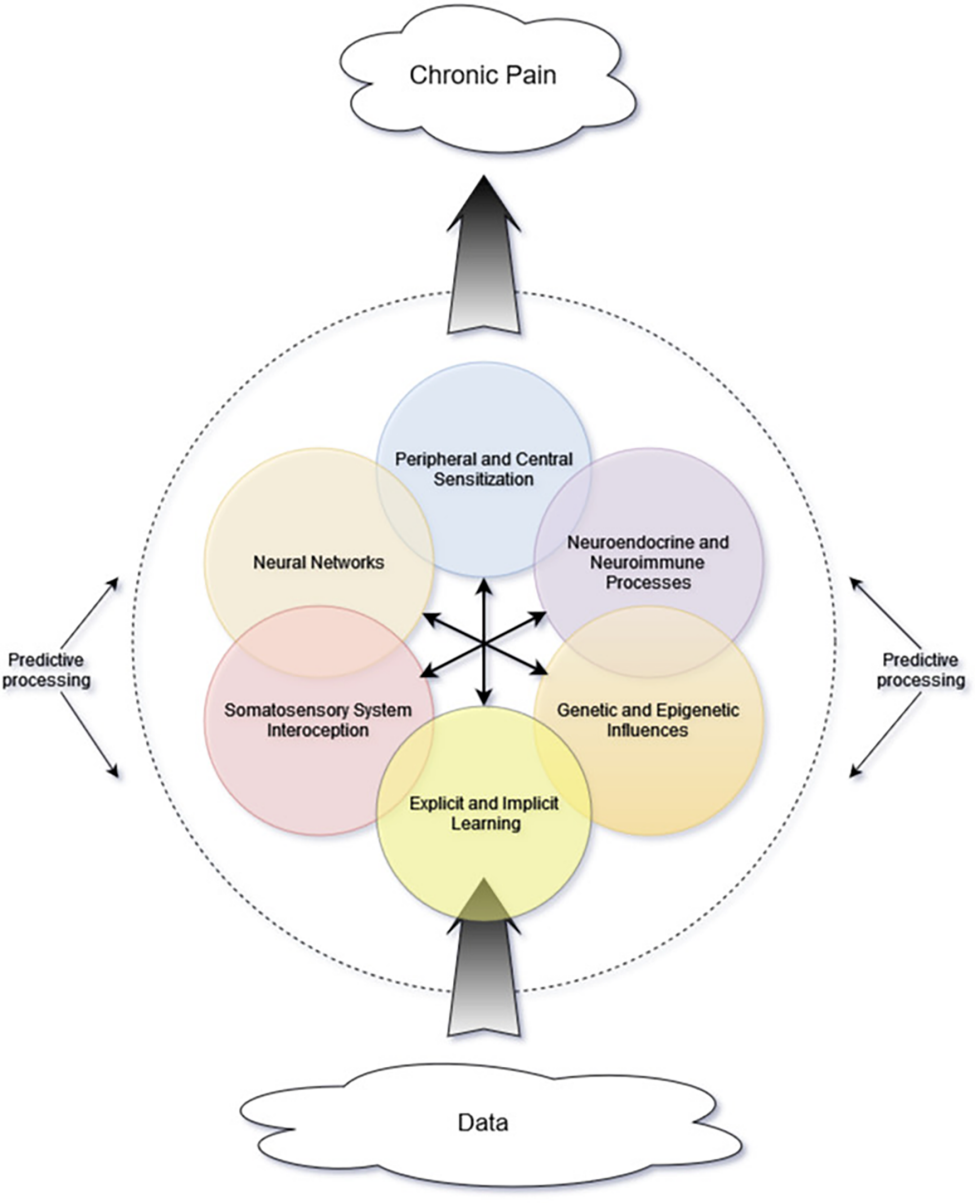
Proposed schematic representing interacting components and mini-systems. Central arrows represent multidirectional interactions among internal components. As incoming data are processed, their influence and interpretation are affected by many system components, including others not depicted in this simple graphic. The brain's predictive processes are depicted as the dashed line encircling the other components, because these predictive processes not only affect interpretation of internal signals but also perception of and attention to incoming data from the environment.

While the previously described multilevel matrix explanatory model of chronic pain indeed has many components, there exists an additional lens of complexity—that of complexity science—which may lend further insight. In a complex system, there must be interactions among the internal components as well as between internal and external influences (42). Importantly, these interactions are not simply mechanical or linear, with one action sequentially triggering the next. Rather, they often are nonlinear and involve feedback loops and multiple energy states. These complex interactions give rise to “self-organization” and “emergent phenomena” (42). Self-organization refers to the tendency of dynamic systems to favor distinct recurring patterns or states. Emergent phenomena result from interactions between the system and its environment and cannot be predicted based on system components alone (42–44). Figure 1 is a simplified representation of many of the interacting components that contribute to the proposed complex system and the emergent property of chronic pain.

### 2.1 Central sensitization

The concepts of peripheral and central sensitization have been used to explain the development of various chronic pain syndromes and specific phenomena such as allodynia. Both peripheral and central sensitization involve increases in membrane excitability, synaptic efficacy, and neural recruitment, leading to amplification of pain signals (45). A growing body of evidence points to the importance of a (dys)regulation or imbalance of the inhibitory and excitatory neurotransmitters, gamma-amino-butyric acid (GABA) and glutamate in different parts of the brain (46–48), which may account for the changes in neuronal sensitivity. Central sensitization initially was postulated to occur as the result of acute injury, which would then lead to “secondary hyperalgesia” (49). It has since been demonstrated in a variety of pain conditions including fibromyalgia, chronic neck pain, osteoarthritis, migraine, irritable bowel syndrome, chronic fatigue syndrome, and pediatric pain syndromes (50–54).

Concurrently, a similar phenomenon may arise from decreased top-down influence of the descending inhibitory pathways. For example, the pregenual anterior cingulate cortex (pgACC) is part of the descending inhibitory system. It communicates with the rostral ACC (rACC), which is known as the “affective division” of the ACC and is involved in the integration of emotion and cognition (55–57). A deficiency in the inhibiting influence of the pgACC over the rACC has been demonstrated in individuals with fibromyalgia (58, 59). Some authors discuss cognitive-emotional sensitization as a component of central sensitization (16), reflecting the importance of such higher-level regulatory components.

Whether initiated from a lower or higher order within the pain matrix, these processes essentially result in the “pain gate” being left open or the gated system otherwise being leaky, resulting in hyperalgesia and allodynia. Questions remain about why either or both these phenomena occur in some individuals but not in others. Many people rehabilitate injuries without developing chronic pain syndromes. Others develop chronic pain with no identifiable antecedent injury. What might cause changes in neural organization or function that may lead to complex pain syndromes?

### 2.2 Interoceptive dysfunction

Interoception involves sensing and registering the body's internal state, and likewise involves many interacting components. This includes afferent signaling (from joints, tissues, and organs) and centrally mediated representation of the internal physiological states (60, 61). Organizationally, at least some afferent interoceptive signals are coupled to specific efferent physiological outputs via homeostatic reflexes (e.g., baroreflex control of blood pressure) and operate beneath conscious awareness (62–64). Such relay pathways primarily involve spinal, vagal, and glossopharyngeal afferents, with multiple levels of processing and integration in the spinal cord and autonomic ganglia (65, 66). Similar to top-down modulation of nociceptive signals, these homeostatic reflexes are subject to inhibition by descending signals from a higher network—in this case, the central autonomic network (CAN) (62). The CAN is a distributed network that includes the insular and medial prefrontal cortices, parts of the amygdala, hypothalamus, and periaqueductal gray (67). It influences autonomic, endocrine, motor, and behavioral responses, reflecting higher-order “allostatic policies” related to adaptive behaviors and survival in an uncertain external environment (68–70).

Interoception is a fundamental function. Not only is continuous interoceptive processing required to maintain homeostasis and to inform motivational states and emotional experiences, but it even is implicated in one's emergent sense of self (68). While many interoceptive processes do not reach conscious awareness (62), much research assessing the relationship between interoception and the pain experience has focused on the most measurable component, *interoceptive accuracy*, which often is assessed by the objective accuracy in detecting one's heartbeat (71). Decreased accuracy in the heartbeat tracking task has been associated with chronic pain in adults (72). Other dimensions of interoception, which may be even more relevant to persistent pain states, include regulatory and attentional aspects of body awareness (73, 74). Perturbations in interoceptive representation, integration, and predictive control can originate at any level of the neuraxis, including those related to higher-order cognitive appraisal and emotional states (62, 73, 75). One may envision how decreased interoceptive accuracy, especially if combined with catastrophic interpretation of sensory signals, may lead to an overinterpretation of pain and greater distress.

### 2.3 Brain networks

The triple network model by Venod Menon (76) brings together the current model of transdiagnostic neuroscience, the DMN, along with the SN and CEN/FPN. Again, the DMN is known as a resting state network (21, 77). It is engaged during metacognitive processes such as self-reflection (78), theory-of-mind (79), and mind-wandering (80) and is considered to be the “highest level of a functional hierarchy” serving as a “central orchestrator or conductor of global brain function” (81). There is overlap between the DMN and the regions of the brain involved with interoception (82). The CEN/FPN, has a central role in a variety of cognitive functions such as working memory, attention, reasoning, planning, and adjusting (83, 84). The CEN/FPN allows for focused attention by filtering out interfering information (85, 86). The SN is involved in detecting and orienting to salient external and internal stimuli, serving an important function in responding to homeostatic demands (87).

According to the Triple Network model by Menon, a variety of psychopathologies can be explained by altered interactions within and among these three cardinal networks (76). Recently, De Ridder and colleagues applied this model to chronic pain (13). They point out that normally the activities of the CEN/FPN and SN are directly correlated with each other, and both networks are anti-correlated to the DMN (88). In fact, the SN seems to act as the “switch,” which shifts the brain from a resting state (DMN-dominant) into a more focused state (CEN/FPN-dominant) in order to respond to salient stimuli (89).

De Ridder et al. propose that various aspects of chronic pain are the result of connectivity changes between the lateral (ascending) pathway of the somatosensory network, and at least one of the aforementioned neural networks (13). For example, increased functional connectivity with the DMN could relate to increased self-identification with pain that often evolves in the setting of chronic pain syndromes. Increased functional connectivity with the SN could potentially result in increased vigilance, rumination, and pain-related anxiety. Lastly, changes in functional connectivity with the CEN/FPN may be associated with cognitive disability, which has been shown in individuals experiencing chronic pain syndromes (13, 90–92). Functional neuroimaging studies of patients with chronic low back pain have demonstrated hyperconnectivity between the primary somatosensory cortex and the DMN (93, 94). In one study, duration of pain was positively correlated with the strength of connectivity between the primary somatosensory cortex and the DMN (93). This observation is consistent with the Hebbian learning model, colloquially described as *neurons that fire together wire together*. Although the details of how and when these network changes occur remain unknown, correlations between pain experience and the activity and connectivity of these neural networks may have profound implications for pain persistence and treatment resistance (13).

### 2.4 The role of stress

#### 2.4.1 Feedback loops

Feedback loops are critical to homeostatic mechanisms and are characteristic of complex systems (42). Multiple feedback loops exist in the above examples. Here, we focus on a feedback loop involving stress, highlighting interactions between multiple internal system components and aspects of the external environment. Stress is a complex concept that encapsulates not only the real or perceived presence of a threat, but also the organism's ability to predict, mitigate, or otherwise adequately respond to that threat (95, 96). Analogous to the acute vs. chronic pain distinction, acute stress may be adaptive, whereas chronic stress appears to be maladaptive and has been implicated in many disease processes and mental health disorders (97).

During the physiologic stress response, cortisol enters the brain and binds to glucocorticoid and mineralocorticoid receptors, which are well represented in the limbic system and the prefrontal cortex. These interactions enable classic gene-mediated immunomodulatory actions of cortisol as well as faster non-genomic actions (24, 98), including effects on kinase signalling pathways (99) and changes in ion transport (100). These latter mechanisms may contribute to the previously described central sensitization by affecting membrane polarization. Ordinarily, cortisol negatively feeds back higher up the HPA axis to regulate its own production, but this feedback is not a simple loop. In addition to direct feedback (which can be altered by changes in receptor density, configuration, and responsiveness), the HPA axis and its response to cortisol also are influenced by complex neurocircuitry involving oligosynaptic networks between limbic structures (e.g., hippocampus, amygdala) and the paraventricular nucleus of the hypothalamus. (101, 102). Chronic stress is associated with alterations in both anatomy and function of multiple parts of the limbic system and its integrating neurocircuitry (102). Various changes in the stress-modulating brain structures (particularly, the hippocampus, amygdala, and prefrontal cortex) and associated circuits can lead to dysregulation of the entire HPA, resulting not only in hypercortisolism or hypocortisolism but also altered cortisol reactivity and changes in anticipatory responses to perceived stressors (103, 104).

Pain and stress exhibit a bidirectional relationship, since both acute and chronic pain are in themselves stressors, signalling actual or perceived danger and potentially triggering the HPA axis, while acute and chronic stress affect the pain experience. For example, acute stress can affect sensitivity to experimentally-delivered noxious stimuli resulting in increased or decreased pain experiences (26, 105–107). Acute stress also can exacerbate pain intensity in the context of chronic pain (108), resulting in pain flares even in the absence of a physical insult.

The relationship between chronic stress and pain is complicated. The corticolimbic system and the thalamus are key regions associated with the endocrine stress response (25, 109) and with pain persistence (110). Although measured cortisol levels do not show consistent patterns in the setting of chronic pain, dysregulation of the HPA axis does seem to be an important component of this complex system (111). Furthermore, chronic stress is associated with anxiety and depression, which also overlap with chronic pain both demographically and mechanistically (112, 113). Indeed, the concept of mutual maintenance focuses on the perpetuation of the interrelated phenomena of stress, chronic pain, poor sleep, and mental health disorders (114–116). The parallels between stress and pain are highlighted by Abdalla and Geha (111), who suggest that they may be “two sides of the same coin.” Although they also point out conflicting data, they propose that “stress and pain can be two nodes in a vicious circle of maladaptive responses to environmental challenges leading to compromised well-being.”

The striking overlap among the above conditions begs the question of a common root cause or predisposing factor that increases risk for HPA axis dysregulation, depression/anxiety, and chronic pain syndromes. A growing body of research suggests early life trauma or adverse childhood experiences (ACEs) may play a role. Since publication of the ACE study by Felitti and colleagues, which showed a dose-responsive correlation between ACE score and multiple adverse health outcomes (117), many investigators have added to an understanding not only of the associations between childhood adversity and clinical and behavioural outcomes but also of the etiologic underpinnings of these relationships, centred around two factors: epigenetics and learning (118–121).

#### 2.4.2 Stress, epigenetics, and pain

Epigenetics refers to persistent changes in gene expression that alter cellular activity and function without alterations in the primary DNA sequence (122). Genes may be up-regulated, down-regulated, or otherwise altered through various mechanisms including methylation and histone modifications affecting chromatin folding and unfolding (both processes acting to regulate transcription) as well as through the effects of small, noncoding RNA and posttranscriptional editing of RNA (affecting translation) (122, 123).

In rodent models, a wide variety of stressors have been employed in attempts to elucidate the mechanisms that translate stress into physiologic and clinical outcomes, including pain conditions. Physical restraint, recurrent unpredictable stress, maternal separation, and even prenatal stressors have been associated with increased sensitivity to visceral pain (124–126). Emerging data suggest that epigenetic changes are important mediating factors (127, 128). Epigenetic changes resulting in transcriptional dysregulation of glucocorticoid-related genes have been linked with the development of several stress-related disorders (122, 129), and prevalence of at least some of these epigenetic changes appears to be increased in individuals with histories of ACEs (130, 131). More recently, it has been demonstrated that even acute trauma in adulthood can affect methylation status of genes involved in the production of adrenocorticotropic hormone (132).

Risk for chronic pain syndromes also appears to be associated with ACE scores (133–135) in humans. Similarly, studies in rodents have shown an association between maternal separation from neonates and the development of pain sensitivity in adolescents (136, 137). Recent data, again in humans, suggest that epigenetic markers might be prognostic for the development of chronic pain states after surgery (138) or other acute trauma (132). These and other epigenetic changes can have long-lasting effects for the individual such that stressful experiences become “biologically embedded” and may even be passed on to offspring, resulting in intergenerational transmission (123). Conservation of stress-induced epigenetic changes across generations has been demonstrated in both mouse and human studies (139–142).

#### 2.4.3 Stress, learning, and pain

Stress responses and chronic pain may become cognitively embedded through learning. Stress and pain engage the learning circuitry of the hippocampus, amygdala, and prefrontal cortex as animals face environmental challenges to homeostasis (143–145). A form of implicit learning that appears to be evolutionarily vital is non-associative conditioning, in which behavior is modified in response to a single event. Behavioral sensitization is a form of non-associative conditioning where aversive or noxious stimuli lead to amplification of defensive behavioral responses (128). Another form of sensitization can be witnessed during critical periods—developmental windows during which certain kinds of learning (e.g., language acquisition) appear to be concentrated (146, 147). During critical periods, perturbations in the physical or social environment may have particularly significant, longer lasting effects on learning and neuroplasticity (148, 149).

The above mentioned brain regions also play critical roles in a process likened to “unlearning,” known as fear extinction or extinction learning (143, 150, 151). Failures in extinction learning have been associated with PTSD and chronic pain (152–156). Furthermore, postmortem and imaging studies show decreased hippocampal volume and other anatomic and functional changes in the brains of these individuals, suggesting the presence of a chronic process that may reinforce and/or perpetuate disorders of learning and memory (157–159). It can be postulated that early life stressors (including those occurring *in utero*) trigger epigenetic changes resulting in increased physiologic sensitivity to homeostatic perturbations; ongoing stressful environments (especially those occurring during critical periods) affect learning, potentially limiting adaptive responses. Under such conditions, it is perhaps unsurprising that additional incidental trauma(s), with or without an associated injury, might precipitate chronic pain. This scenario invokes another concept in complexity science, nonergodicity. Nonergodicity refers to the influence of initial conditions (in this case: prior conditions and associated epigenetic modifications) on the trajectory of response following a perturbation to the system (42). Such changes may provide fertile ground for the emergence of nociplastic pain,, the management of which requires a bio-psycho-social approach (160).

## 3 The brain as a prediction machine

### 3.1 Introduction to Bayesian theory and the free energy landscape

When chronic pain is understood as an emergent property within a complex adaptive system, Bayesian theory, hierarchical coding, and computer learning become valuable tools for garnering insights relevant to clinical medicine. Indeed, these are beginning to be used to model key features of chronic pain syndromes. Although computer modeling is beyond the scope of this article, a brief discussion may illuminate certain cognitive-behavioral components relevant to the development and management of chronic pain syndromes.

A foundational paper by Thomas Bayes in the late 18th century first applied what would become Bayesian theory to gambling and games of chance (161). It is based on the premise that information, upon which decisions are made, often is incomplete. For any situation requiring a decision, there can be hidden or unobservable elements, and available information may be vague, imprecise, or irrelevant (also termed “noisy”). Consequently, a decision invokes probabilities based upon past experiences (“priors”) and perceived degrees of certainty surrounding the data. Once the outcome of the decision is observed or experienced, probabilities can be updated to inform future decisions. Although assessing and updating probabilities is largely subconscious, the process is iterative and informs our evolving beliefs and expectations (162, 163).

The application of Bayesian theory to mental health emerged over the last several decades as researchers and clinicians recognized its potential in understanding and managing complex conditions. Some have leaned heavily on Bayesian theory along with the free energy principle to propose transdiagnostic explanations for a wide range of mental health conditions as well as chronic pain (164–167). Understandably so, since many hidden factors contribute to the pain experience, and much of the interoceptive and environmental data related to pain is ambiguous, incomplete, or otherwise noisy and requires inference for interpretation.

The free energy principle, applied to complex systems, explains behavior as an adaptive response to uncertainty (168). It posits that living systems have an inherent tendency to resist disorder and to minimize uncertainty (163). Most organisms live in an ever-changing environment and are faced with moment-to-moment challenges with variable levels of predictability. To survive and thrive, all organisms must create order. Physiologically, this is called homeostasis, which, just like any other entropy-fighting effort, requires energy. Free energy represents a measure of how well the organism's internal states align with its expectations or predictions (163, 169). It is the overall level of uncertainty in the organism's internal and external states. According to the free energy principle, living systems behave to minimize it.

Closely related to free energy is prediction error, which indicates a discrepancy between the organism's expectation and what it actually perceives in any given interaction (163, 170). Prediction errors are the building blocks of free energy. To minimize free energy, one must minimize prediction errors. Much of modern neuroscience likens our human brains to “prediction machines” or “inference generators.” Our brains are constantly making predictions and inferences about most everything. Therefore, minimizing prediction error is critical to efficient navigation of, and function within, our environments.

To minimize prediction errors, when the brain's prediction does not match incoming sensory information, either the brain revises its prediction, so its belief matches sensory input, or it signals the body to act in a way such that new sensory input matches the pre-existing belief (163, 165). The latter strategy, known as active inference, may occur through sensory attenuation or enhancement or through shifts in attention (170). Both strategies, (revising predictions and active inference) have been proposed to function in various interoceptive processes, including “interoceptive learning” (171, 172).

Using predictive coding, which is based on Bayesian theory and the free energy principle and incorporates machine learning, Anna-Lena Eckert and her group at the University of Marburg propose that, “chronic pain emerges when prior beliefs and likelihoods are biased towards inferring pain from a wide range of sensory data that would otherwise be perceived as harmless” (165). By manipulating input variables, their computerized predictive coding model reproduced key features of chronic pain experiences: hyperalgesia, allodynia, and chronicity. This model assumes that (1) pain perception emerges from the Bayesian combination of sensory input and prior beliefs, (2) chronic pain involves maladaptive learning processes over longer timescales, (3) these learning processes result in generalized and heightened expectations of pain as well as (4) greater likelihood to erroneously interpret harmless data as dangerous or painful (165).

The model's behavior is affected by manipulation of several variables, largely related to pain expectation and the degree of rigidity vs. flexibility of attribution with respect to both the cause of the sensation and belief in its persistence. In this model, the “chronic pain observer” is characterized by heightened prior expectations of pain, aberrant associations between sensory inputs and pain perception, and heightened assumptions about pain persistence. Each time a given sensation is interpreted as pain reinforces a prior belief, “leading to a stabilization of the system within this pathological state” (165). This stabilization means the presence of conflicting innocuous sensory stimulation does little to influence the inferred state of pain—and is an example of the self-organization that occurs in complex systems. The predictive coding model demonstrates that over time each iteration is “less and less likely to infer a pain-free state from any type of incoming sensation.” Supporting empirical data suggest that individuals with persistent pain syndromes may attend less to present sensory information and rely more heavily on their prior expectations of pain (173, 174).

### 3.2 Rigidity and canalization

The above computer-modeled process and its outcomes align with a mechanism known as canalization. The term was first used in developmental biology to describe the tendency of developmental processes to be resistant to external or internal perturbation and to be relatively constrained to particular trajectories (175). Recently, canalization has been proposed by Carhart-Harris and colleagues to underlie many psychopathologic conditions (164). [Also see: (176, 177)]. The group's general theory states that “cognitive and behavioral phenotypes that are regarded as psychopathological are canalized features of mind, brain, or behavior that have come to dominate an individual's psychological state space.” These canalized features may develop for a variety of reasons, and the depth of entrenchment largely determines the severity of the psychopathology, including the degree of treatment-resistance and susceptibility to relapse.

Similarly, Fabio Giommi and colleagues focus on rigidity (cognitive-behavioral inflexibility) as a core feature of psychopathology, noting that one of the primary domains in which rigidity may play a role concerns the self, including inflexible identity narratives, limiting self-beliefs, and habitual patterns of behavior. Importantly, decreasing rigidity may be important for restoring and maintaining mental health (178). The group cites evidence (179) that while several transdiagnostic processes are associated with psychological distress and disability, it is rigidity that “makes these transdiagnostic processes problematic and eventually pathologic,” that is, under limited volitional control.

Although both groups briefly mention chronic pain syndromes as having canalized or rigid elements, neither group fully explores the relevance of these concepts or their proposed models to the field of chronic pain. We and other authors (111, 179, 180) propose that chronic pain syndromes share many characteristics with disorders categorized as “internalizing” phenotypes which include negatively biased cognitions and avoidance (181, 182). Indeed, there is a great deal of overlap among chronic pain, depression, and anxiety disorders (10, 112, 183), and high rates of comorbidity among these conditions strongly suggest the need for transdiagnostic approaches.

Different types of rigidity may have roles in chronic pain. For example, physical rigidity is not only a result of physical processes like inflammation, scarring, or joint space narrowing, but also avoidance and alterations of movement. Restricted behavioral patterns may be motivated by fear of pain, which is itself perpetuated by negative reinforcement learning characterized by cognitive-behavioral-emotional rigidity or canalization.

In canalized phenotypes, belief-updating fails to occur (181), and a person's thoughts and behaviors become “entrenched in overly precise beliefs” and may become tightly bound to schemas of self (173). For many people who have lived with persistent pain for years, pain sensations and associated distress become inseparable from their daily experience and an influential factor in how they define themselves and navigate the world. Such a coupling between the pain experience and self-identity may be mediated through increased functional connectivity between the insula and other parts of the interoceptive network and the DMN. This canalization process and resultant stability of pathologic features have been reproduced in the computer-learning model by Eckert's group for chronic pain specifically (165). More generally, canalization is at the heart of a theory proposed by Robin Carhart-Harris, Karl Friston, and others (164) explaining how observed neurobiological effects of psychedelics may be relevant to their therapeutic applications to mental health conditions.

### 3.3 Decanalization: A potential role for psychedelic therapy

Carhart-Harris et al. defined the REBUS model (RElaxed Beliefs Under pSychedelics) (81), in which psychedelics disrupt the normal hierarchical modus operandi of the brain, characteristic of ordinary consciousness. Psychedelics are described as agents that increase entropy in spontaneous cortical activity related to normal cognitive-perceptual processes. The relaxation of beliefs facilitated by psychedelics is proposed to occur secondary to reduced influence of “pathologically overweighted priors” (i.e., rigid “rules” and associated beliefs and behaviors that develop in response to painful or distressful experiences). Overweighted priors heavily influence the Bayesian process and may lead to various expressions of mental dysfunction and illness and, according to our argument, may likewise feature prominently in the development and maintenance of chronic pain. De-weighting of said priors presumably allows for relaxed assumptions and beliefs that were previously constrained, fostering a more flexible mindset (81, 164, 184, 185).

REBUS is informed by dynamic systems theory, which translates complexity science into adaptive approaches for addressing complex problems. In dynamic systems theory, all possible states of a given system—usually called the “phase space”—are visualized as an undulating landscape of low-lying attractor-basins, repellent peaks, and all the various trajectories in between (81, 186). Overweighted priors create seemingly immovable features, such as hills and mountains that dominate the landscape and affect the dynamic trajectories of inputs, just as the flow of rain and water is affected by the contours of a natural landscape. If raindrops represent incoming bottom-up sensory data, one can visualize how the flow of this interoceptive information could be directed—canalized—into cognitive behavioral patterns (streams and rivers) that can become more and more entrenched over time.

Another analogy used by Carhart-Harris and colleagues (81, 187) describes the entropic effects of psychedelics on the brain. They liken this to the process of metallurgy, in which the addition of heat energy can transform a once rigid solid into a material that is more flexible and changeable. If the psychedelic experience can undo or reduce the overemphasis of rigidly held beliefs through the disruptive increase in entropy (analogous to heat), the result may be a “flattened” (decanalized) landscape and an opportunity to rebalance the topography such that new patterns may emerge. The effects of this process are likely to be “felt most profoundly when at the highest or deepest levels of the brain's functional architecture such as those related to selfhood, identity, or ego” (81).

Of course, the perturbation of increased entropy alone does not guarantee that a healthy, balanced landscape will emerge. Take, for example, the devastation that can occur with earthquakes or, more relevant to psychopathology, strokes or severe prolonged psychotic episodes. To increase the chances for a therapeutic effect, other energy and information must be presented and integrated into the system as it settles and cools. It is well established that certain “extra-pharmacological factors” including preparation, intention-setting, and integration are important to outcomes in psychedelic-assisted therapy (PAT). These inputs are regarded as significant additive factors for positively reshaping the free-energy landscape and reducing the negative influence of formerly dominant thought patterns and behaviors. The importance of the combination of a psychedelic compound with specific contextual elements is the premise on which PAT is based (188–190). When done thoughtfully and within a safe, supportive therapeutic relationship, PAT may be able to catalyze a transformative process and accomplish what years or decades of therapy alone could not—or so say many therapists who already are working adjunctively with medicines such as ketamine and recent promising research efforts involving MDMA or psilocybin. This may perhaps arise because, as Carhart-Harris and Friston state, “precision weighting of high-level priors must be *relaxed* before they can be *revised* (italics added)” (81).

### 3.4 The free-energy landscape and energetic economics

Two of the three previously mentioned large-scale neural networks—the SN and CEN/FPN—have roles in both conscious and unconscious learning (191–193). Although the DMN does not seem to be involved in focused, effortful learning, it may contribute to unconscious learning through reinforcement and perpetuation of habitual thought patterns and repetitive, self-focused mental activity (194). As presented previously, changes in functional connectivity between the somatosensory cortex and the cardinal networks of the DMN, SN, and CEN/FPN may explain various characteristics associated with chronic pain syndromes (13). Although not the first group to provide evidence of these changes, De Ridder and colleagues went a step further to provide a reason as to why they may occur: energetic economics.

The brain represents only 2% of total body weight yet accounts for more than 20% of the total daily energy requirements in humans (195). Though modern humans rarely lack adequate caloric intake, throughout most of human history, energy sources were less ubiquitous and predictable. Therefore, as modern human brains were evolving, so too were many strategies for conserving energy. One of those strategies may be partly responsible for the chronicity of a variety of mental health disorders as well as chronic pain syndromes.

The sympathetic and parasympathetic divisions of the autonomic nervous system greatly affect energy distribution and overall energy requirements. Activation of the sympathetic system increases energy consumption (196, 197), whereas a parasympathetic-dominant state conserves energy. The SN, which responds to novel stimuli relevant to homeostasis, overlaps with the sympathetic control network of the brain (13, 198), whereas the DMN overlaps with the parasympathetic network (13, 199).

High-salience settings of threat often involve acute pain and/or fear. In these acute situations, daily energy expenditure is increased as much as 60% (13, 200). With such high costs, maintenance of these sympathetic states is energetically prohibitive. However, in chronic pain and anxiety states, energy expenditure is increased to a far lesser degree (176, 177). De Ridder and colleagues propose that “energy expenditure could be reduced by rewiring the pain pathways to connect to the default mode network, which overlaps with the parasympathetic central network and disconnect from the energy-consuming sympathetic nervous system” (13).

Mounting evidence shows that functional connectivity within and between these three cardinal networks is abnormal in numerous brain disorders including depression, anxiety, and posttraumatic stress disorder (76). Studies on resting-state networks and their functional connectivity in patients with chronic pain syndromes [including migraine (201), fibromyalgia (59), and complex regional pain syndrome (93, 202)] also have shown disrupted network properties, including failure to deactivate core regions of the DMN (203) [but see (204) for a different perspective]. The longer the pain persists, the more the connections between the primary somatosensory cortex and the DMN may be strengthened (93). This strengthened relationship between the pain experience and the self-representational DMN may explain why pain can become an integral part of the self-percept. Meanwhile, the dorsal anterior cingulate cortex (dACC), which is a central hub of the sympathetic nervous system, appears to be relatively deactivated in chronic pain compared to acute pain (199). Therefore, despite potentially contributing to pain chronicity, there may be an energetic advantage to this arrangement.

To summarize, the free-energy landscape of the Bayesian brain may become dominated by relatively inaccessible peaks (established by e.g., trauma), well-worn paths of cognitive-emotional canalization, and attractor basins resulting from energetic economics, which predict a tendency to remain in the lower energetic points of the landscape. Translating this to real-life experiences of millions of people living with diverse forms of chronic pain: Many interacting factors, often unknown or unconscious to the individual are at play as the brain attempts to make sense of ambiguous sensory data and associated environmental and contextual information. The emergent subjective pain experience is often emotionally charged and may threaten one's sense of safety or even one's place in the world. The Bayesian brain uses past experiences to inform probabilities about the source and intensity of the pain, its likely duration, and its relevance to our lives. Past experiences themselves arose in the context of “noisy” sensory and socioenvironmental inputs requiring interpretation, have since been consolidated through emotion-charged learning, and are recalled through imperfect memory that is influenced by present-time stress and emotional state. Under these conditions individuals tend to reify ideas about a particular source of pain (e.g., via imaging studies that report “degenerative discs,” or “bone on bone arthritis,” or via remote injuries), about what triggered the pain [“the last time I (did X), I suffered for a week”], and about what has or has not helped in the past (“physical therapy just made things worse!”). Fears about the permanence of the pain that can be triggered through diagnostic jargon, as well as unsatisfying interactions in both medical and broader social contexts, are layered into this landscape, contributing to chronicity. Beneath such higher order cognitive Bayesian interpretations, epigenetic changes may contribute to the sensitization of one's nervous and endocrine systems, and to inflammation/neuroinflammation, adding fuel to the fire. Anxiety and/or depression (whether present at baseline or developed in response to chronic pain and associated stress) reinforce negative biases cementing in canalized beliefs and behaviors that perpetuate physical and mental discomfort. Putting it all together, is it any wonder that narrowly focused therapies based on reductionist principles are largely ineffective in the setting of chronic pain?

## 4 Expanded rationale for the use of psychedelic therapies for chronic pain

### 4.1 Psychedelics as disruptive agents

Certainly, it is not feasible to identify and intervene at every aberrant interaction within this complex system. Instead, disrupting the system's landscape while simultaneously introducing targeted, corrective therapies may have rippling effects modulating multiple components and altering interactions within the system. Such an intervention may indeed have the greatest chance at meaningful, lasting improvements in the chronic pain experience. Psychedelic therapies seem to exhibit such properties and may therefore be poised as potentially useful treatments for chronic pain.

The word psychedelic is derived etymologically from Greek roots for “mind or soul” and “clear or manifest,” combining to “mind-manifesting,” coined by British psychiatrist Humphry Osmond in 1957. Put simply, these are substances that may reveal qualities of mind that were previously unknown in ordinary conscious awareness. Classic serotonergic psychedelic compounds are those with hallucinogenic effects mediated primarily through the 5-hydroxytryptamine (5-HT) 2A receptor, and include psilocybin (and its active metabolite psilocin), mescaline, lysergic acid-N,N-diethylamide (LSD), and N,N-dimethyltryptamine (DMT) (205). Other compounds that share some potentially therapeutic effects and may have overlapping mechanisms of action but interact with different receptors include 3,4-methylenedioxymethamphetamine (MDMA), ibogaine, and the dissociative anesthetic ketamine. For the purpose of our discussion, the remainder of this paper focuses on classic serotonergic psychedelics unless otherwise specified.

Although REBUS is a theoretical model, there is mounting evidence in support of its premise. Neuroimaging studies show that classic psychedelics and other consciousness-altering substances such as ketamine and MDMA reliably affect the DMN (206, 207). For example, functional magnetic resonance imaging (fMRI) studies of individuals under the influence of psychedelics show acute decreases in connectivity and blood flow within nodes of the DMN (208, 209). This reduced functional connectivity has been associated with states of ego dissolution often occasioned by these substances (210, 211). At the same time, connectivity between the DMN and other brain networks increases, reducing functional segregation (207, 212–214), which may explain not only the bizarre perceptual experiences elicited by psychedelics but also the experiences of insight reported by participants in clinical trials of psilocybin and other psychedelics (187, 215–217). In their review article, Gattuso and colleagues conclude that “there are clear associations between a psychedelic's ability to reduce the functional connectivity within the DMN (and increase its connectivity to other networks), altered states of consciousness, and therapeutic outcomes” (206).

Beyond these intra- and internetwork trends, existing imaging studies reveal very heterogeneous effects of psychedelics, making it difficult to draw conclusions about their potential therapeutic mechanisms. This task was recently undertaken by Girn and colleagues, who propose that “the brain under psychedelics is best seen as entering a distinct mode of functioning, which can be characterized as being more dynamically flexible, diverse, and tuned for global information sharing” (216). In addition to the increased entropy of spontaneous brain activity (81), this temporary mode of functioning has the capacity to completely disrupt the former free-energy landscape. When paired with personalized therapy to prevent or minimize a return to previous canalized patterns, the altered brain activity facilitated by psychedelics may be a powerful catalyst for deep and lasting transformation.

### 4.2 Psychedelics as stimulators of neuroplasticity

A unique aspect of psychedelic therapies, demonstrated in clinical trials as well as observational studies, is that beyond the profound acute effects that occur during exposure, there often are enduring therapeutic effects that persist long after the psychedelic compounds are fully metabolized (218–222). Many of these longer-term effects likely are related to the increased neuroplasticity that appears to be a downstream effect of the activity on 5HT2A and other receptors in the brain (223–225).

Neuroplasticity underlies the brain's ability to change, which occurs across the lifespan (226). Neuroplastic changes occur on molecular, cellular/subcellular, and functional levels and include the phenomena of dendritogenesis and synaptogenesis [for reviews, see (227, 228)]. The details of how psychedelics stimulate neuroplastic change are still being uncovered, but preclinical and clinical studies demonstrate increases in molecular and structural neuroplasticity after single doses of psilocybin, LSD, and Ayahuasca/DMT (222, 229, 230). Some of these effects appear to be mediated through activation of the 5-HT2A receptor and can be blocked by 5-HT2A receptor antagonists (231). However, preclinical research demonstrates 5-HT2A receptor-independent neuroplasticity in mice (222, 232, 233), suggesting that multiple pathways may be involved. Indeed, at least some classic psychedelics also activate the tyrosine kinase receptor 2 (TrkB) for brain-derived neurotrophic factor (BDNF) (232)—as do ketamine (234) and MDMA (222, 235).

The abilities of these agents to induce neuroplastic changes may be of particular relevance in nociplastic pain syndromes, which involve changes in function along the neuraxis, resulting in phenomena such as central sensitization in the absence of ongoing tissue damage. These changes themselves represent neuroplasticity in a manner that heightens sensitivity to incoming pain-relevant sensory signals and affects higher-order interpretation and learning (45, 236). While learning and neuroplasticity occur throughout the lifespan, recall that certain kinds of learning (e.g., social learning in mice, song-learning in birds, language acquisition and visual development in humans) are concentrated during critical periods. During critical periods, animals’ brains are more sensitive to relevant environmental signals, enhancing learning until the window closes. Interventions that might keep the window open longer or reopen it after it has been closed have been of great interest to neuroscientists and clinical researchers. Recent data show that serotonergic and other psychedelics (MDMA, ketamine, and ibogaine) can reopen critical-period social learning in mice (233). Should these results translate to humans, the implications for learning could be profound.

There also are implications for unlearning, as neuroplasticity is also required to unlearn and effectively reprogram the nervous system to respond to stimuli in more adaptive ways. As introduced previously, extinction learning may be particularly important for persistent pain conditions. Animal models using different psychedelic substances show improvements in fear-extinction learning (237–239), enhanced memory performance (240–242), strengthened cortico-hippocampal synapses (222, 243), and improvements in clinical correlates of anhedonia, anxiety, and learned helplessness (238, 243, 244). In humans, psychedelic-associated cognitive flexibility has been described as enhanced cognitive reappraisal (245, 246), creative thinking (247), insight into personal problems, emotional breakthroughs, and reprocessing of traumatic memories (215, 246, 248–250). Multiple studies also show increased personality trait openness following psychedelic experiences (251–253). This increase in trait openness is associated with decreased depression and anxiety (254–257) and is of particular interest since the big five personality trait factors have long been assumed to be relatively immovable (258).

### 4.3 Meeting complexity with complexity

Our current understanding of mechanisms underlying the therapeutic actions by psychedelic therapies is far from complete, but it can be postulated that these diverse molecules and their downstream effects, including priming the brain for concurrent or subsequent interventions, may be as complex as the conditions we hope to treat. Most of the preceding discussion is similarly relevant to depression, anxiety, PTSD, eating disorders, and a variety of other conditions. Beyond these observations of their effects on neural connectivity, at least some psychedelic compounds also have physiological effects that may mitigate various components of the pain matrix (259, 260). In some cases, these effects may not require mind-altering subjective experiences associated with psychedelics and are active areas of pharmaceutical research and development.

#### 4.3.1 Systemic inflammation and neuroinflammation

The serotonin receptor subtype 5-HT2A appears to be critical for the psychedelic-defining effects of the classic psychedelics. The 5-HT2A receptor is one of the most widely expressed serotonin receptors in the body and has been found in nearly every tissue and cell type examined (261, 262). This receptor appears to be involved with a great many processes, including mediating pro-inflammatory effects of serotonin (263). Drugs that antagonize the 5-HT2A receptor can block inflammation (264), so it might be expected that agonists, such as the classic psychedelics would increase inflammation. However, psychedelics may instead stabilize the 5-HT2A receptor conformation in such a way that favors anti-inflammatory signaling (265). This may explain the findings by Nichols and colleagues, who showed that the compound (R)-2,5-dimethoxy-4-iodoamphetamine [(R)-DOI], a 5-HT2A agonist that induces typical psychedelic effects at high doses, has impressive anti-inflammatory effects at low (subperceptual) doses in mice. Their group found that the anti-inflammatory effects of this and several classic psychedelic agents were not correlated with the respective behavioral potencies (e.g., head twitch response, considered to be an indicator of above-threshold “psychedelic” effects in mice) (262). Moreover, unlike corticosteroids, which have problematic broad-spectrum immunosuppressive effects, the psychedelic compounds studied suppressed key proinflammatory biomarkers but never below baseline levels, “leaving the immune system largely intact but affecting enough to normalize physiology” (262).

With respect to neuroinflammation, recall that microglia are the primary immune cells in the brain. Microglia appear to be intimately involved in regulating neuroplasticity and the inflammatory environment of the brain (266). Neuroinflammation has been associated with pain syndromes such as migraines and cluster headaches (267, 268) as well as depression (36, 269, 270) and cognitive complaints associated with Lyme disease (271, 272). Migraine alone affects approximately 38 million people in the US and approximately 1 billion people worldwide (273). Published reports regarding the benefits of LSD for individuals with migraine first appeared in the 1960s (274). More recently, the first randomized controlled trial of psilocybin for the management of migraines found significant reduction in frequency and intensity of migraines after a single dose (275). Individuals with cluster headaches also report benefit associated with intentional use of psychedelic compounds, including LSD and psilocybin. In a survey conducted by the advocacy group, Clusterbusters, patients ranked the preventive effectiveness of psilocybin significantly above that for verapamil and prednisone, two conventional treatments used preventively in headache syndromes (276).

Although the effects of classic psychedelics on microglia have yet to be fully elucidated, existing literature suggests an anti-inflammatory effect, potentially paralleling research on peripheral immune cells (277). In addition to 5-HT2A receptor modulation, at least some psychedelics may interact with the sigma-1 receptor, expressed on microglia and which controls ATP synthesis in the mitochondria (278). It appears that subjective psychedelic effects are not requisite for systemic anti-inflammatory effects, as demonstrated by Nichols et al., nor for therapeutic benefit in headache disorders. Even 2-bromo-LSD, a congener of LSD with greatly reduced psychotropic effects, appears beneficial in early clinical trials (279, 280). Such insights are relevant for advancing mechanistic understanding and to clinical feasibility for potential applications of psychedelic compounds in the context of pain.

#### 4.3.2 Nociception and analgesia

Given the complex nature of pain, concepts of nociception and analgesia may need to broaden. This aside, in this section we focus on molecular signaling within the current definition of the pain neuraxis, an area of research with predominantly pharmaceutical applications.

There are fourteen known 5-HT receptors in humans, several of which may be involved in peripherally and centrally mediated pain processes. The 5-HT2A and 5-HT7 receptors are involved in pain inhibition and exert different effects in acute vs. chronic pain states (260). Other research has identified roles for 5-HT1 (281, 282) and 5-HT3 (283). In a rodent model of neuropathic pain, allodynia and hypersensitivity were induced with chemotherapy in wild-type mice but not in 5-HT2A receptor knockout mice (284), suggesting a role for the receptor in the development of central sensitization. In a separate rat study, the allodynia and hypersensitivity were reversed with the selective 5-HT2A antagonist, glemanserin (284). As with the anti-inflammatory example, the apparent paradox of using a 5-HT2A agonist as an antinociceptive agent may be explained by receptor stabilization (285, 286), internalization (287), or partial agonism [reviewed in (205)]. Furthermore, chronicity and tissue- and region-specificity may affect receptor response to ligand binding (260). Again, with classic psychedelics much attention has been paid to 5-HT2A, but it appears that psilocin (the active metabolite of psilocybin) binds to nearly all 5-HT receptor subtypes with affinity similar to or greater than its affinity for 5-HT2A receptors (288, 289), leaving open a wide area of research related to downstream effects.

In a recent study using a rat model of chronic pain, a single dose of intravenous psilocybin resulted in significant attenuation of mechanical (although not thermal) hypersensitivity (290). Human clinical studies of psychedelic treatments in pain conditions are sparse, with most published data coming from anecdotal reports and survey-based studies. For example, Bonnelle and colleagues report on responses from 250 individuals who self-identified as having chronic pain and who had experience with psychedelics, either in large doses (reaching hallucinogenic threshold, aka “macrodoses”) or sub-threshold doses (i.e., “microdoses”), or both. They found self-reported pain relief with low or high doses was statistically superior to nonsteroidal anti-inflammatories, but only macrodosing was statistically superior to opioids or cannabis. Furthermore, the duration of effect was longer after macrodosing than after microdosing (291). At the time of this writing, the only published results from a randomized controlled trial was that of Ramaekers and colleagues, who showed that a single dose of LSD in healthy subjects resulted in reduced acute pain response to the cold-pressor test (292). There are a number of clinical trials of psychedelic therapies in pain syndromes currently in progress, including those for fibromyalgia, chronic low back pain, phantom limb pain, and headache syndromes. The results of these studies will hopefully enhance our understanding and generate more research questions.

#### 4.3.3 Sleep

Sleep disturbances are another common comorbid complaint among people with chronic pain as well as those with depression and anxiety disorders, and several models have been proposed in which sleep disruption represents a shared underlying mechanism of these common and potentially debilitating conditions (15, 293–295). Sleep affects memory consolidation (296), information processing (297), fear extinction (298), and emotional regulation (299, 300). When depression and insomnia co-occur with chronic pain, they are associated with worsening of many pain outcomes (115, 301–303). Although there are limited data about sleep interventions on clinical outcomes in those living with chronic pain, a 2021 systematic review concluded that improved self-reported sleep quality and quantity seemed to be associated with decreased pain and disability (304).

Sleep is also one of the biological processes proposed to contribute to the persistent therapeutic effects seen after psychedelic interventions (305). Serotonergic psychedelics have been shown to affect sleep patterns (306–308), and the 5-HT2A receptor is known to play a role in sleep (307). In addition, an open-label study found that a one-time dose of ibogaine was associated with improvements in sleep in veterans with PTSD (309). It has been proposed that the dissociative anesthetic ketamine may affect circadian rhythms through the downregulation of “clock” genes, potentially contributing to its antidepressant effects (310). To our knowledge, there have been no studies specifically assessing the relationship between sleep and pain after psychedelic therapies.

### 4.4 Combining modalities to optimize clinical outcomes

While it appears that psychedelic therapies may affect the pain experience in many ways—from nociception to inflammation to higher-level cognitive-emotional effects and reopening of critical periods—it is likely that the optimization of clinical outcomes will require a multimodal approach incorporating other transdiagnostic treatments. Multimodal interventions already are recommended for the management of chronic pain; psychedelic therapies may facilitate synergistic effects through their effects on neuroplasticity, mood, mindset, sleep, and other factors.

**Figure 2:**
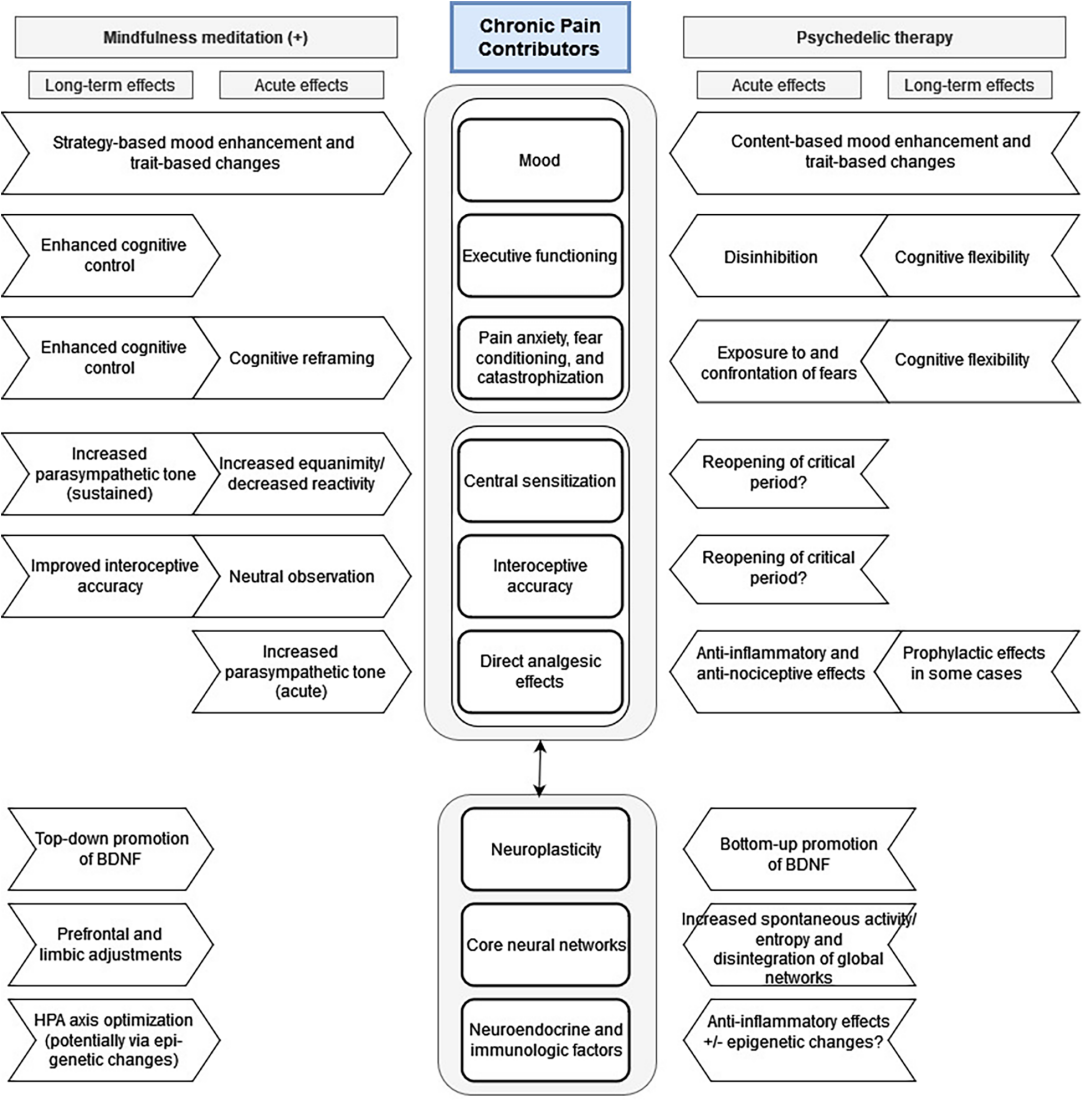
Proposed mechanisms for acute and long-term effects of psychedelic and mindfulness therapies on chronic pain syndromes. Adapted from Heuschkel and Kuypers: *Frontiers in Psychiatry* 2020 Mar 31, 11:224; DOI: 10.3389/fpsyt.2020.00224.

Prior to the so-called “psychedelic renaissance,” much attention was being paid to mindfulness meditation and other “third wave” therapeutic approaches, which have been shown to have several effects that are similar to those seen with PAT. Specifically, several meditation and mindfulness practices have been shown to improve cognitive flexibility and neuroplasticity (311–316). In a 2020 review paper by Kristin Heuschkel and Kim P.C. Kuypers, the authors synthesized a large amount of research into an excellent comparison of mindfulness meditation and psychedelic therapies, emphasizing how the two may complement each other, potentially resulting in synergistic beneficial effects related to mood and anxiety states as well as executive and social functioning (317). As these factors likewise contribute to the chronic pain experience, we adapted their model [ Figure 2 in (317)] and included additional factors particularly relevant to pain: central sensitization, interoceptive accuracy, and direct analgesic effects (Figure 2).

As discussed, there is a growing body of research related to the anti-nociceptive and anti-inflammatory properties of classic psychedelics and non-classic “psychedelic adjacent” compounds such as ketamine and MDMA. The mechanisms may vary depending on the compound and its receptor affinities and likely differ from those of mindfulness/meditation practices. The latter have been shown to have short-term analgesic effects (318, 319) as well as longer-term effects on sympathetic/parasympathetic tone and neuroendocrine reactivity to stressors, including pain (320, 321). With respect to acute pain, the analgesic effect seen with mindfulness meditation does not appear to be mediated by endogenous opioids, demonstrated by the lack of reversal of these effects by the opioid antagonist naloxone (322).

Mindfulness practices also affect major neural networks, including the DMN. For example, higher trait mindfulness, which can be increased through mindfulness-based mental training, was associated with greater deactivation of the posterior midline nodes of the DMN (323). Mindfulness and other forms of meditation also have been shown to modulate the insula and other parts of the interoceptive network (172). Indeed, interoceptive learning, as defined by García-Cordero and colleagues (171) relies on neuroplastic changes invoked by interoceptive training, which can include mindfulness practices (172). Interoceptive feedback influences emotional states and cognitive processes (75, 324, 325), is closely linked to the nociceptive and affective systems (62) and may form the cortical basis for selfhood and self-awareness (75, 326), the latter which can be profoundly affected in psychedelic-induced states. Should there be a critical period or periods for interoceptive learning, psychedelics may be able to reopen those periods, potentially enhancing interoceptive accuracy.

Current excitement about neuroplasticity may be warranted despite being poorly understood. To assess neuroplasticity in humans, researchers rely either on imaging to evaluate grey matter changes or on measurement of BDNF. Preclinical models have also relied on indirect assessment by BDNF or by direct assessment of dendritogenesis and synaptogenesis via microscopic analysis of brain tissue. With these methods, a variety of treatments for depression, including traditional antidepressants (327), transcranial magnetic stimulation (328), electroconvulsive therapy (327, 329), exercise (330, 331), and acute sleep deprivation (332) have been shown to stimulate neuroplasticity. Psychedelics are among the most effective chemical modulators of neural plasticity studied to date (333). Enhanced neuroplasticity as measured by BDNF levels in human volunteers also has been shown to occur with prolonged, repeated practice of various forms of meditation (317, 334).

Not explicitly included in Figure 2 but certainly relevant to both MDD and chronic pain is the role of insight with respect to understanding recurring cognitive, emotional, and behavioral patterns. The phenomenology of insight and its relationship with meditative practices as well as psychedelic therapies was recently reviewed by Tulver and colleagues (335), who state that “insight occurs as a novel understanding when previous problematic mental representations about the self and others are restructured or overwritten by new knowledge structures.” Both mindfulness meditation (336, 337) and psychedelic experiences (246, 338, 339) appear to facilitate insight, which may result in part from the “decentering” effects (e.g., disruption of the DMN) and which likely contributes to therapeutic effects of both modalities.

## 5 Conclusions

While conventional reductionist approaches may continue to be of value in understanding specific mechanisms that operate within any complex system, chronic pain may deserve a more complex—yet not necessarily complicated—approach to understanding and treatment. Psychedelics have multiple mechanisms of action that are only partly understood, and most likely many other actions are yet to be discovered. Many such mechanisms identified to date come from their interaction with the 5-HT2A receptor, whose endogenous ligand, serotonin, is a molecule that is involved in many processes that are central not only to human life but also to most life forms, including microorganisms, plants, and fungi (261). There is a growing body of research related to the anti-nociceptive and anti-inflammatory properties of classic psychedelics and non-classic compounds such as ketamine and MDMA. These mechanisms may vary depending on the compound and the context within which the compound is administered. The subjective psychedelic experience itself, with its relationship to modulating internal and external factors (often discussed as “set and setting”) also seems to fit the definition of an emergent property of a complex system (216).

Perhaps a direction of inquiry on psychedelics’ benefits in chronic pain might emerge from studying the effects of mindfulness meditation in similar populations. Fadel Zeidan, who heads the Brain Mechanisms of Pain, Health, and Mindfulness Laboratory at the University of California in San Diego, has proposed that the relationship between mindfulness meditation and the pain experience is complex, likely engaging “multiple brain networks and neurochemical mechanisms… [including] executive shifts in attention and nonjudgmental reappraisal of noxious sensations” (322). This description mirrors those by Robin Carhart-Harris and others regarding the therapeutic effects of psychedelics (81, 216, 326, 340). We propose both modalities, with their complex (and potentially complementary) mechanisms of action, may be particularly beneficial for individuals affected by chronic pain. When partnered with pain neuroscience education, movement- or somatic-based therapies, self-compassion, sleep hygiene, and/or nutritional counseling, patients may begin to make important lifestyle changes, improve their pain experience, and expand the scope of their daily lives in ways they had long deemed impossible. Indeed, the potential for PAT to enhance the adoption of health-promoting behaviors could have the potential to improve a wide array of chronic conditions (341).

The growing list of proposed actions of classic psychedelics that may have therapeutic implications for individuals experiencing chronic pain may be grouped into acute, subacute, and longer-term effects. Acute and subacute effects include both anti-inflammatory and analgesic effects (peripheral and central), some of which may not require a psychedelic experience. However, the acute psychedelic experience appears to reduce the influence of overweighted priors, relaxing limiting beliefs, and softening or eliminating pathologic canalization that may drive the chronicity of these syndromes—at least temporarily (81, 164, 216). The acute/subacute phase of the psychedelic experience may affect memory reconsolidation [as seen with MDMA therapies (342, 343)], with implications not only for traumatic events related to injury but also to one's “pain story.” Finally, a window of increased neuroplasticity appears to open after treatment with psychedelics. This neuroplasticity has been proposed to be responsible for many of the known longer lasting effects, such as trait openness and decreased depression and anxiety, both relevant in pain, and which likely influence learning and perhaps epigenetic changes. Throughout this process and continuing after a formal intervention, mindfulness-based interventions and other therapies may complement, enhance, and extend the benefits achieved with psychedelic-assisted therapies.

## 6 Future directions

Psychedelic-assisted therapy research is at an early stage. A great deal remains to be learned about potential therapeutic benefits as well as risks associated with these compounds. Mechanisms such as those related to inflammation, which appear to be independent of the subjective psychedelic effects, suggest activity beyond the 5HT2A receptor and point to a need for research to further characterize how psychedelic compounds interact with different receptors and affect various components of the pain neuraxis. This and other mechanistic aspects may best be studied with animal models.

High-quality clinical data are desperately needed to help shape emerging therapies, reduce risks, and optimize clinical and functional outcomes. In particular, given the apparent importance of contextual factors (so-called “set and setting”) to outcomes, the field is in need of well-designed research to clarify the influence of various contextual elements and how those elements may be personalized to patient needs and desired outcomes. Furthermore, to truly maximize benefit, interventions likely need to capitalize on the context-dependent neuroplasticity that is stimulated by psychedelic therapies. To improve efficacy and durability of effects, psychedelic experiences almost certainly need to be followed by reinforcement via integration of experiences, emotions, and insights revealed during the psychedelic session. There is much research to be done to determine what kinds of therapies, when paired within a carefully designed protocol with psychedelic medicines may be optimal.

An important goal is the coordination of a personalized treatment plan into an organized whole—an approach that already is recommended in chronic pain but seldom achieved. The value of PAT is that not only is it inherently biopsychosocial but, when implemented well, it can be therapeutic at all three domains: biologic, psychologic, and interpersonal. As more clinical and preclinical studies are undertaken, we ought to keep in mind the complexity of chronic pain conditions and frame study design and outcome measurements to understand how they may fit into a broader biopsychosocial approach.

In closing, we argue that we must remain steadfast rather than become overwhelmed when confronted with the complexity of pain syndromes. We must appreciate and even embrace this complex biopsychosocial system. In so doing, novel approaches, such as PAT, that emphasize meeting complexity with complexity may be developed and refined. This could lead to meaningful improvements for millions of people who suffer with chronic pain. More broadly, this could also support a shift in medicine that transcends the confines of a predominantly materialist-reductionist approach—one that may extend to the many other complex chronic illnesses that comprise the burden of suffering and cost in modern-day healthcare.

## Data Availability

The original contributions presented in the study are included in the article/supplementary material, further inquiries can be directed to the corresponding author.
